# Effectiveness of Cinnamon Oil Coating on K-wire as an Antimicrobial Agent against Staphylococcus Epidermidis

**DOI:** 10.5704/MOJ.1311.010

**Published:** 2013-11

**Authors:** R Magetsari

**Affiliations:** Orthopaedics Department, Sardjito Hospital, Yogyakarta, Indonesia

## Abstract

Background: Chronic osteomyelitis remains one of the common problems with the use of orthopaedic implants. Staphylococcus epidermidis is notorious for its biofilm formation on indwelling medical devices and is one of the most frequent pathogenic agents in chronic osteomyelitis. Cinnamon oil has been proven to be an effective antimicrobial agent against several bacteria, including S. epidermidis. The eradication of S. epidermidis and prevention of biofilm formation on medical devices are desirable outcomes. Objective: To study the antimicrobial effect of cinnamon oil coating on K-wire against S. epidermidis and to quantify the most effective concentration of cinnamon oil coating on the K-wire. Method: The cinnamon oil was divided in ten different concentrations, from 0.002% to 1%, and subsequently applied to the Kirschner wire (K-wire). Its antimicrobial effect was determined by agar well diffusion method (MHA). Cinnamon oil coated K-wires were planted on S. epidermidis inoculated Muller-Hinton Agar (MHA) plate. The size of the zone of inhibition was recorded to the nearest mm, and this was compared to gentamycin, fosfomycin, vancomycin, netilmycin. Result: The cream based 1% concentration cinnamon oil coating on K-wire showed the strongest antimicrobial effect on S. epidermidis inoculated MHA plate. This was evident especially in the fourth repetition, with an inhibition zone diameter (IZD) of 19 mm. In the 1% concentration repetitions, the highest mean IZD of the 4 repetitions was 14 mm (intermediate according NCCLS). The mean IZD results demonstrate that cinnamon oil has 46.3% of the effectiveness of gentamycin, 49.1% of fosfomycin, 59.6% of vancomycin, and 43.4% of netilmycin. Conclusion: In this in-vitro study, cream based cinnamon oil coating on K-wire is effective against S. epidermidis, though less effective compared to gentamycin, fosfomycin, vancomycin and netilmycin

**Key Words:**

Cinnamon oil, K-wire, antimicrobial, S.epidermidis

## Backgroud

In recent years Staphylococcus epidermidis (S. epidermidis) has become the main pathogenic agent in nosocomial infections and severe sepsis. This is especially in the immune-compromised patients and patients with medical devices and implants in the body. Devices and implants such as cerebrospinal shunts, central venous catheters, heart valve prostheses, contact lenses, intraperitoneal catheters and orthopaedic prostheses are often implicated^1^. Treatment of S. epidermidis infections is increasingly problematic because clinical isolates have shown resistance to an increasing number of antimicrobial agents and more importantly, because of the ability of S. epidermidis to grow as a biofilm. Biofilm formation by S. epidermidis is governed in part by the production of polysaccharide intercellular adhesin 21.

Biofilm formation is an important factor in the pathogenicity of S. epidermidis. This is the mechanism by which the bacteria becomes attached and colonize biomaterials and devices 4. Previous studies have demonstrated that microorganisms within biofilms are less susceptible to antimicrobial treatment than their planktonic counterparts 2, probably due to a combination of poor antimicrobial penetration, nutrient limitation, adaptive stress responses, induction of phenotypic variability, and persister cell formation 15. Current research is focused on identifying new compounds that may have antimicrobial activity against microorganisms, both in the planktonic and biofilm modes.

Plant essential oils have been used in food preservation, pharmaceutical therapies, alternative medicine, and natural therapies for thousands of years 13,23. Cinnamon oil is such an essential oil commonly used in the food industry because of its special aroma 3. Cinnamomum is a genus in the family Lauraceae. Many species are used as spices, one of which is Cinnamomum burmannii from Indonesia, also called Indonesian cassia (the commercial name is “cinnamon stick”). Several publications have demonstrated the antibacterial activity of cinnamon oil isolated from the bark of this species^7^,^10^,^12^, ^26^. Cinnamon oil has also been shown to be effective against biofilm cultures of Streptococcus mutans and Lactobacillus plantarum^9^. Essential oil derived from the leaves of another closely related species within this plant family, Cinnamomum osmophloeum (native to Taiwan), has an excellent inhibitory effect on planktonic cultures of nine gram-positive and gram-negative bacteria, including methicillin-resistant Staphylococcus aureus and S. epidermidis 3.

Previous studies have reported that the predominant active compound found in cinnamon oil was cinnamaldehyde 23,27. Cinnamaldehyde causes inhibition of the proton motive force, respiratory chain, electron transfer, and substrate oxidation, resulting in uncoupling of oxidative phosphorylation, inhibition of active transport, loss of pool metabolites, and disruption of the synthesis of DNA, RNA, proteins, lipids, and polysaccharides^6^,^8^,^20^. In addition, an important characteristic of volatile oils and their components is their hydrophobicity, which enables them to partition into and disturb the lipid bilayer of the cell membrane, rendering them more permeable to protons. Extensive leakage from bacterial cells or the exit of critical molecules and ions ultimately leads to bacterial cell death 23.

The susceptibility of S. epidermidis to cinnamon oil derived from the bark of Cinnamomum burmannii when coated onto K-wire, however, has not been published. The current in vitro study was undertaken to establish the efficacy of cinnamon oil coating on K-wire as an antimicrobial agent against S. epidermidis isolated. Gentamycin, fosfomycin, vancomycin, and netilmycin were used as comparisons.

## Materials and Methods

Bacterial isolates. Bacterial isolates of S. epidermidis, obtained from blood, cerebrospinal fluid, pus, and urine, were collected from Sardjito Hospital, Yogyakarta, Indonesia, and identified in the Microbiology Department, Gadjah Mada University, Yogyakarta, Indonesia 17. The S. epidermidis were cultured on nutrient agar medium and incubated at 37°C for 24 hours. One hundred microlitres (100 μl) of standardized inoculums (106 CFU/ml; 0.5 MacFarland) of bacterium was spread with the help of sterile spreader onto sterile Muller-Hinton Agar (MHA) (Hi-Media) so as to achieve confluent growth.

Antimicrobials. Cinnamon stick (Cinnamomum burmannii), produced in Indonesia, was obtained from a local market in Tawangmangu in central Java, Indonesia, and subjected to authentification by botanical experts. Cinnamon oil was extracted by steam distillation to obtain the volatile oil 26. Stock solutions of 16% cinnamon oil in 5% propylene glycol (PG) was prepared. Cinnamon oil was emulsified on a cream base in ten different serial concentrations (ranging from 0.002% to 1%).

Determination of antimicrobial activity of cinnamon oil coated on K-wire. Each of the ten different concentrations of the cinnamon oil on cream base emulsification was coated on K-wire of diameter 2 mm and length 10 mm. Each of the cinnamon oil cream bases coated K-wire was individually planted on Muller-Hinton Agar and incubated in 37°C for 24 hours. Subsequently, the cream base in ten different serial concentrations was introduced in quadruplicates into the agar plates. These served as controls. Gentamycin disc, fosfomycin disc, vancomycin disc and netilmycin disc were also included in the study. The zone of inhibition was recorded to the nearest 1 mm in size 19.

The results were expressed in terms of the diameter of the inhibition zone: ≤12 mm, resistance; 13 – 14 mm, intermediate; ≥ 15 mm, sensitive^16^. These were compared to the diameter of the inhibition zones of gentamycin, fosfomycin, vancomycin, and netilmycin.

## Results

### Inhibitory activity against bacteria

Cinnamon oil coating on K-wire was found to have an antibiotic effect against S. epidermidis. The strongest antibiotic effect of the cinnamon oil cream base coating on K-wire on S. epidermidis was shown at a concentration of 1% during the fourth repetition where the IZD of 19 mm was graded as “sensitive” based on the NCCLS criteria (Table I). The highest average IZD of 14 mm was shown by the 1% concentration of Cinnamon oil cream base on 4 repetitions. This was graded as having an “intermediate effect” according to the NCCLS criteria (Inhibition zone diameter: ≤ 12 mm, resistance; 13-14 mm, intermediate; ≥ 15 mm, sensitive)(Table II).

The fourth repetition of cinnamon oil cream base coating on K-wire was found to be 67.9% of the effectiveness of gentamycin, 61.3% of fosfomycin, 82.6% of vancomycin, and 59.4% of netilmycin. The mean result of all 4 repetitions of cinnamon oil cream base coating on K-wire was found to be 46.3% of the effectiveness of gentamycin, 49.1% of fosfomycin, 59.6% of vancomycin, and 43.4% of netilmycin.

## Discussion

The activity of cinnamon is attributed to the presence of cinnamaldehyde, an aromatic aldehyde that inhibits amino acid decarboxylase activity^29^. This has been proven to be active against many pathogenic bacteria 28. Cinnamon bark is rich in cinnamaldehyde (50.5%), which is highly electronegative. Such electro-negative compounds interfere in biological processes involving electron transfer and they react with nitrogen-containing components such as proteins and nucleic acids, thereby inhibiting the growth of microorganisms. Cinnamon oil contains benzoic acid, benzaldehyde and cinnamic acid. The lipophylic moiety of these compounds has been recognized as being responsible for its antimicrobial property^24^. Also, cinnamon oil from bark contains 4.7% eugenol 25. Members of this class are known to be either bactericidal or bacteriostatic agents, depending upon the concentration used 22. These compounds have been found to be strongly active despite their relatively low capacity to dissolve in water 5,11,14. Essential oil from cinnamon bark also contains cinnamyl acetate (8.7%), which increases the activity of the parent compound.

Several studies have investigated the antimicrobial effects of cinnamon oil 3,9,23,27. In a previous study, cinnamon oil has been shown to have antimicrobial activity against both planktonic and biofilm cultures of clinical S. epidermidis strains 18. However, the effect cinnamon oil coating on Kwire against S. epidermidis, either in planktonic or biofilm cultures have not been previously reported. The synergistic activity of cinnamon oil with other antimicrobial agents has also been reported. Our results have shown that the cinnamon oil cream base coating on K-wire is against S. epidermidis.

When compared with gentamycin, vancomycin, fosfomycin and netilmycin, cinnamon oil in cream base has a lower antibiotic effect. This may be explained by the unequal spread of oil in the cinnamon oil in cream base when applied on the S. epidermidis implanted MH agar medium, while the spread of Gentamycin, Vancomycin, Fosfomycin and Netilmycin were equal because of the antibiotic discs used in these comparison antibiotic studies.

The findings in this study suggest that cinnamon oil when used to coat orthopaedic implants may reduce the occurrence of chronic osteomyelitis related to orthopaedic implant usage.

**Table I:**
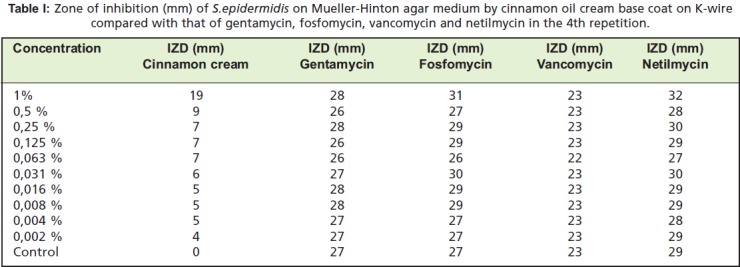
: Zone of inhibition (mm) of S.epidermidis on Mueller-Hinton agar medium by cinnamon oil cream base coat on K-wire compared with that of gentamycin, fosfomycin, vancomycin and netilmycin in the 4th repetition.

**Table II:**
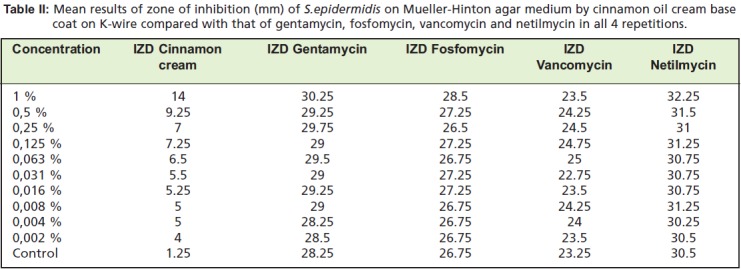
: Mean results of zone of inhibition (mm) of S.epidermidis on Mueller-Hinton agar medium by cinnamon oil cream base coat on K-wire compared with that of gentamycin, fosfomycin, vancomycin and netilmycin in all 4 repetitions.

**Picture 1:**
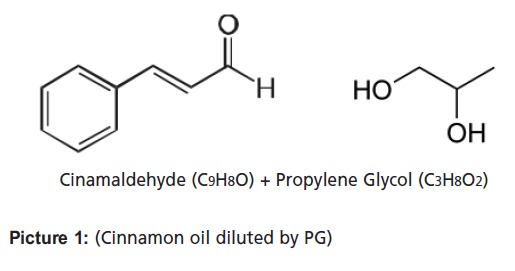
: (Cinnamon oil diluted by PG)

**Picture 2:**
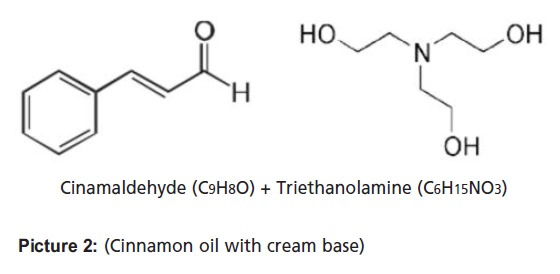
: (Cinnamon oil with cream base)

## Conclusion

In conclusion, this study demonstrates that cinnamon oil in cream base when coated onto K-wire has an antimicrobial activity against clinical S. epidermidis. This may represent a possible alternative method of using a naturally occurring antimicrobial substance as a coating in orthopaedic implants to prevent growth of bacteria, thereby reducing the rate of orthopaedic implant related infections. Further research into the bioadhesive properties of cinnamon oil is needed. The surfaces of the orthopaedic implants in question may need to be reassessed to improve its coating properties with the cinnamon oil.
